# Diagnosis and treatment of deep neck abscess due to congenital piriform sinus fistula in children

**DOI:** 10.1016/j.bjorl.2019.12.008

**Published:** 2020-01-25

**Authors:** Jing Bi, Xiaowei Chen, Zhiying Zhou, Bin Xu, Yong Fu

**Affiliations:** Children's Hospital of Zhejiang University School of Medicine, Department of Otolaryngology Head and Neck Surgery, Hangzhou, China

**Keywords:** Congenital piriform sinus fistula, Deep neck abscess, Children, Diagnosis, Treatment

## Abstract

**Introduction:**

Congenital piriform sinus fistula is a relatively rare type of disease in clinical practice, most occurring during childhood, but doctors have insufficient knowledge regarding this disease, easily misdiagnosing it.

**Objectives:**

This study aimed to identify the characteristics of deep neck abscess due to congenital piriform sinus fistula in children.

**Methods:**

We performed a retrospective study of 21 cases from January 2016 to August 2018 in our hospital. The onset age, clinical characteristics, auxiliary examination and clinical treatment of the patients was summarized to analyze the diagnosis, treatment characteristics and prognosis.

**Results:**

Children from 11 days to 12 years-old were enrolled, with an average age of 3.5 years. Twenty patients had left congenital piriform sinus fistula and 1 had right congenital piriform sinus fistula. Cervical enhanced computed tomography imaging showed gas-liquid equilibrium or air-shadow in the abscesses in 18 cases, and neck ultrasound demonstrated gas echo in the thyroid region in 10 cases. All patients underwent low temperature plasma to seal the internal fistula and returned to the hospital for electronic laryngoscope and neck ultrasound examination at 3 months, 6 months and 1 year after the surgery. No recurrence occurred in any patient.

**Conclusion:**

Congenital piriform sinus fistula is an important cause of deep neck abscess in children. The presence of purulent gas-liquid equilibrium or air shadow in cervical-enhanced computed tomography or ultrasound suggests a high possibility of the presence of an internal fistula, and endoscopic low temperature ablation can be done at the same time as the diagnostic endoscopy.

## Introduction

Congenital piriform sinus fistula (CPSF) is a congenital cervical sacral deformity that is caused by incomplete degeneration of third and fourth cleft palate during early embryonic development. This CPSF deformity accounts for about 1%–10% of all sacral deformities. More than 90 % of patients had this deformity in the left neck, and more than 80 % occurred during childhood.^1^, ^2^ The sinus that opens in the piriform fossa is considered as the most common type clinically. Most of these types have a history of upper respiratory tract infection, and often present as a deep lower neck abscess or acute suppurative thyroiditis.^3^, ^4^ This is a relatively rare type of infection in clinical practice; doctors commonly have insufficient knowledge regarding this disease, misdiagnosing it easily. A repeated neck puncture and drainage is usually applied along with large doses of antibiotics. Due to repeated stimulation of acute or chronic inflammation and surgical drainage, the scar hyperplasia obviously occurred on the neck, causing serious physical and psychological damage to children. This study intends to retrospectively analyze the data of 21 children with deep neck abscess due to CPSF from Children's Hospital of Zhejiang University School of Medicine, summarize the diagnosis and treatment characteristics and prognosis, and improve the physician’s understanding of children's CPSF.

## Methods

### Patients

This retrospective study included consecutive patients who visited either the clinical department for the therapy of deep neck abscess or the emergency department for the therapy of severe upper airway obstruction from January 2016 to August 2018. Of these 21 children, 15 were males and 6 were females, with age ranging from 11 days to 12 years, and an average age of 3.5 years old. The onset time was 1–6 days, and the average time was 2.5 days. This retrospective study was approved by the Ethics Committee of Children's Hospital of Zhejiang University School of Medicine (nº 2019-IRB-112).

### Data collection

**Figure 1:**
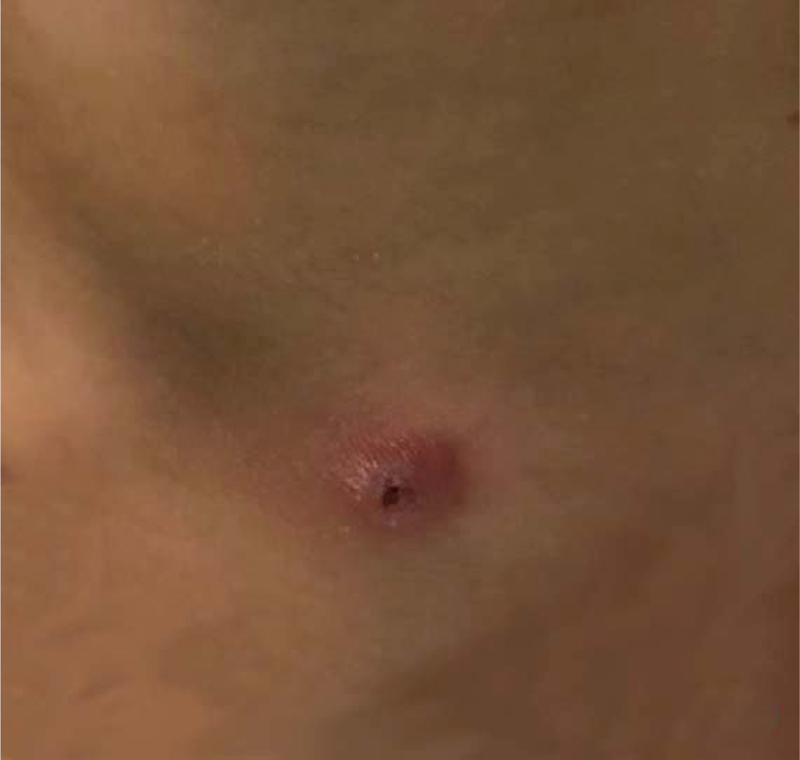
External mouth on the left neck, with peripheral skin congestion.

Clinical information was obtained through a retrospective medical chart review, including symptoms, physical examination, laboratory examinations, and auxiliary examinations. In 14 cases, the symptoms were observed for the first time before seeking medical advice; in 4 cases a repeated left neck bulge was observed (in which one showed improvement after every antibiotic treatment, and external fistula on the left neck was visible) (Fig. 1); 3 cases had cervical scar hyperplasia due to repeated drainage in other hospitals; and 12 cases had a history of upper respiratory tract infection (Table 1).

**Table 1:** Clinical features and auxiliary examination of 21 children with deep neck abscess.

Category	Number	First symptom	Laboratory examinations			Auxiliary examinations	
			WBC (×10^9^/L)	CRP (mg/L)	Thyroid function	Enhanced CT	Ultrasound
Peri-thyroid abscess	14	Anterior cervical mass Sore throat, fever	18.6 ± 3.1	31.3 ± 2.6	Normal	Peri-thyroid abscess and gas shadow (11/14, Fig. 2)	Hypoemic mass (5/14). Gas echo inthe upper left thyroid region (9/14, Fig. 3)
Posterior pharyngeal abscess	4	Lower neck mass II difficulty breathing, refuse to feed	28.6 ± 3.6	57.1 ± 6.6	Normal	Post-pharyngeal abscess and air shadow or gas-liquid level (Fig. 4)	Hypoemic mass
Parapharyngeal abscess	3	Lower neck mass, III degree dyspnea	20.5 ± 2.7	43.2 ± 6.3	Normal	Parapharyngeal space abscess (Fig. 5)	Hypoemic mass

**Figure 2:**
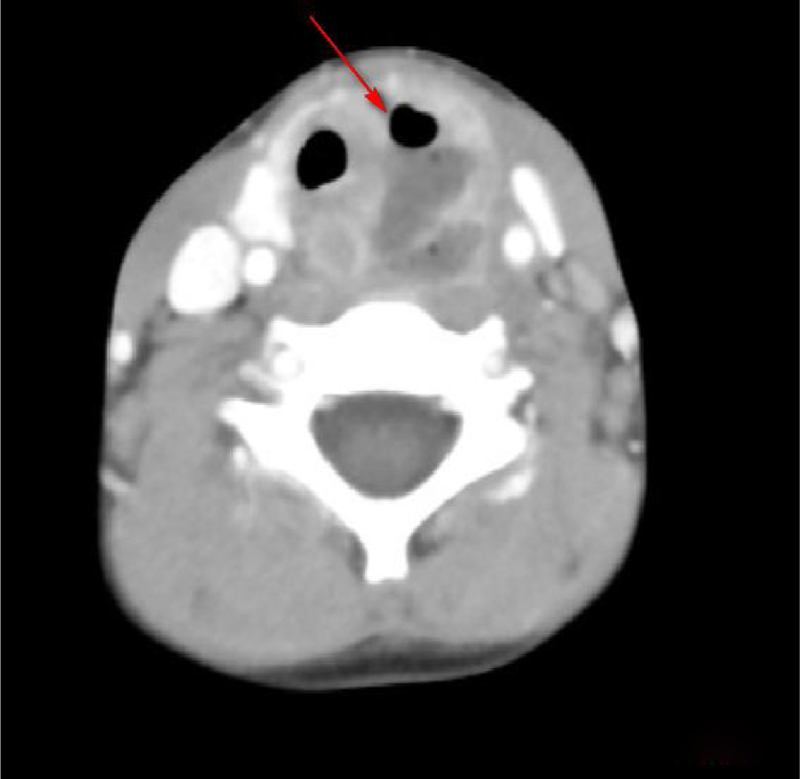
Enhanced CT: left peri-thyroid abscess and gas shadow.

**Figure 3:**
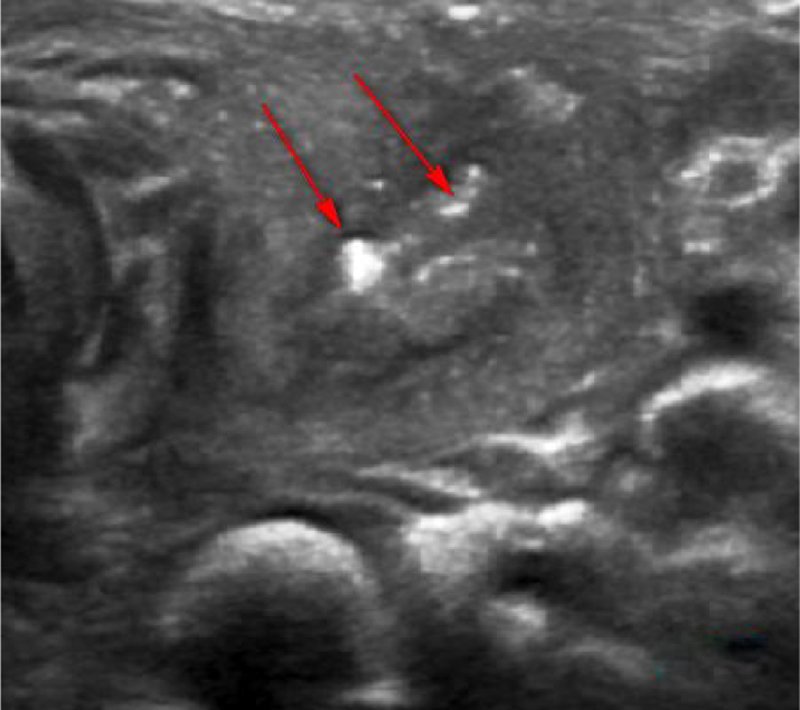
Ultrasound examination: strong echoes of gas scattered in the left thyroid abscess.

**Figure 4:**
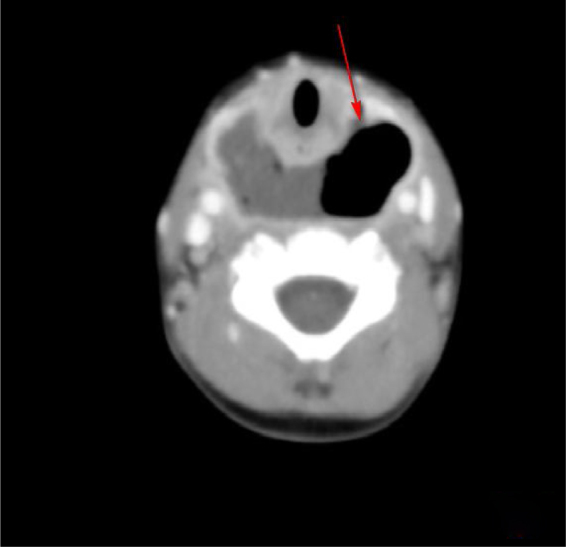
Enhanced CT: Post-pharyngeal abscess and air shadow (Horizontal scan).

**Figure 5:**
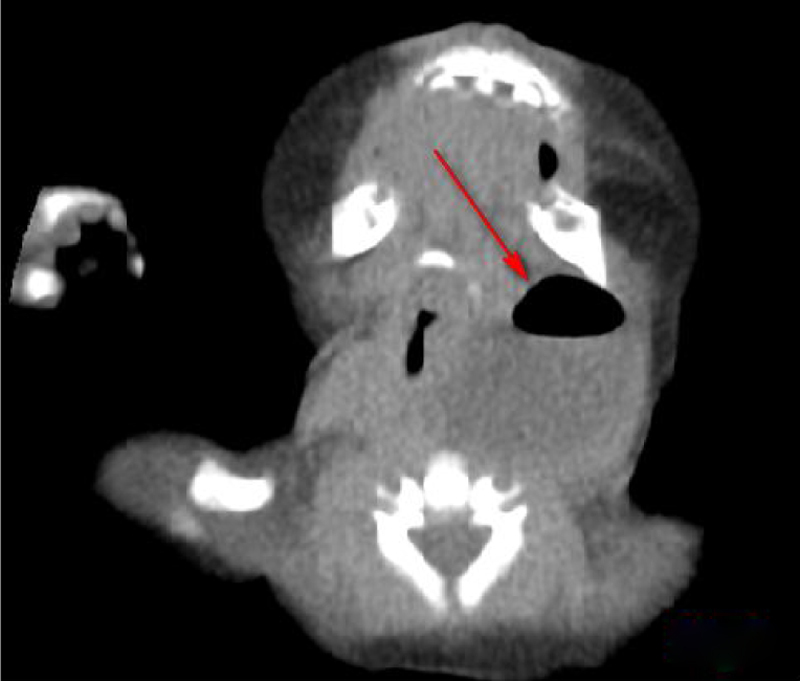
Enhanced CT: Parapharyngeal space abscess and air shadow (Horizontal scan).

### Treatment

**Figure 6:**
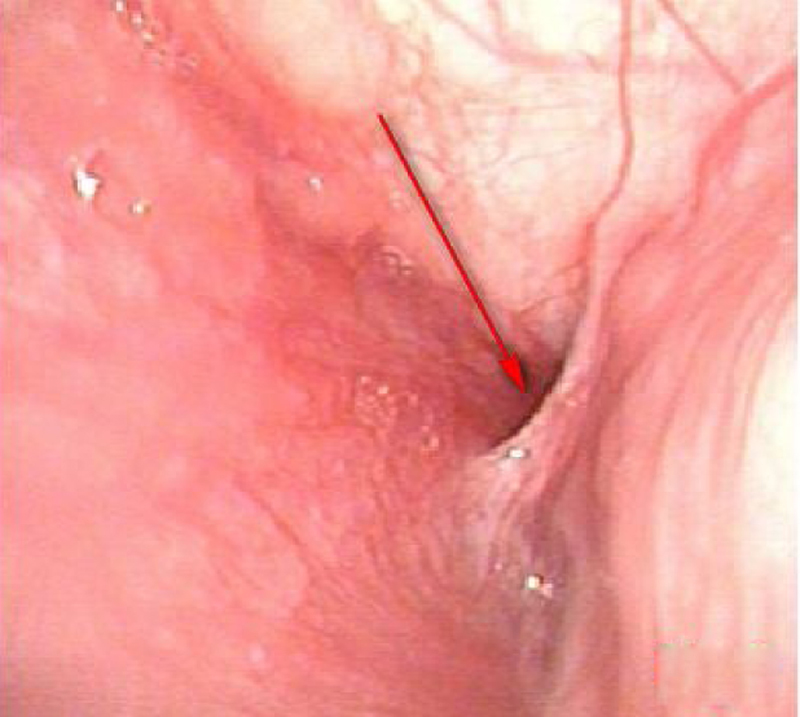
Presence of an internal of left piriform fistula under a self-retaining laryngoscope.

**Figure 7:**
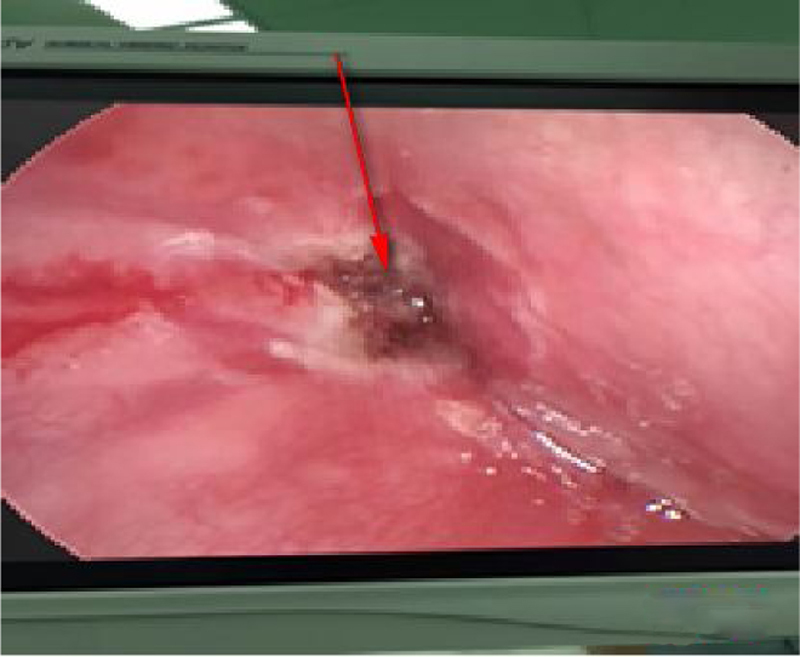
The left piriform sinus fistula after burning under a self-retaining laryngoscope.

All children were treated with antibiotics after hospitalization. Drainage was performed under anesthesia after the formation of abscess liquefaction. At the same time, the piriform fossa was exposed under a self-retaining laryngoscope during operation to check for the existence of a fistula. If an internal fistula was present (Fig. 6), then a low temperature plasma (Smith & Nephew, EIC7070-01) was performed to cauterize it (Fig. 7).

Surgical equipment include a self-retaining laryngoscope and 0 ° nasal endoscope. The patient's teeth should be protected when the affected side of the piriform fossa is exposed by a self-retaining laryngoscope, and adjusted at low temperature plasma of the stall 3 to ablate the parietal fossa tube wall. To protect the recurrent laryngeal nerve, the depth of ablation should be controlled to generally not more than 1.5 mm. Continuous ablation time was 1–2 s, each ablation interval was 2 s, and the number of operations was 2–3 times. Meanwhile, the temperature was quickly perfused with a large amount of ice water during the operation. The neck was pressed onto the opposite side during the operation to prevent accidental injury of anterior lateral laryngeal artery. The self-retaining laryngoscope was removed when no bleeding existed.

A drainage tube was placed in the neck incision according to the size of the abscess, while a gastric tube was placed for 1 week. The dressing was changed daily, and a more effective antibiotic was instituted according to the results of bacteria in the pus culture and drug sensitivity test. Moreover, all patients were given systemic nutritional support. Patients were followed up at 3 months, 6 months and 1 year after the surgery. The laryngeal condition was observed by electronic laryngoscope. Also an ultrasound was performed.

## Results

Of the 21 patients, 20 were diagnosed with left CPSF by self-retaining laryngoscopy, while one was diagnosed with right CPSF. Fourteen cases were diagnosed and dealt with by surgical treatment for the first time in our hospital, and meanwhile, 7 cases developed repeated abscess formation. All children were discharged after the surgery with a cure rate of 100%: the treatment duration ranged 7–65 days.

There were 7 cases with newly diagnosed parapharyngeal space abscess and posterior pharyngeal abscess. All these patients were less than 4 years old and showed an airway obstruction when hospitalized. After providing tracheal intubation, they were able to breathe easily. Drainage was performed under general anesthesia. An indwelling drainage tube was included with daily dressing change before discharging from the hospital. Because of cervical swelling or breathing difficulties, they were all re-hospitalized for 14 days to 2 months after the previous surgery. When they were under general anesthesia for undergoing drainage again, the piriform fossa was exposed with the self-retaining laryngoscope, and the internal fistula was located to confirm the CPSF. After that, the internal fistula was cauterized under the guidance of an endoscope.

All patients returned to the hospital for receiving electronic laryngoscopy and neck ultrasound examination at 3 months, 6 months and 1 year after the surgery. No complication and recurrence occurred.

## Discussion

Deep neck abscess refers to the abscess formed after tissue infection of deep fascia in the neck. Compared to adults, children have small pharyngeal cavities, loose connective tissues in the deep neck, rich lymphoid tissues, and poor immunity. Infection most likely causes abscess, and this easily spreads directly or indirectly between the tissues. At the same time, symptoms of systemic poisoning and serious complications might develop due to absorption of necrotic substances, which are considered life-threatening.^5^ Suppurative neck lymphadenitis, foreign body infection, tonsil, pharyngeal and odontogenic infections, sacral cysts and fistulas are common causes of deep neck abscess in children. Sputum cysts and fistulas are congenital diseases. Misdiagnosis easily occurs due to their low incident rates.

CPSF consists of third and fourth cleft palate, with a fistula opening at the base of the piriform fossa, and obliquely inside or outside the ankle joint, and requires piercing the circumflex muscle or inferior pharyngeal muscle along the larynx. The recurrent nerve descends outside the larynx and terminates in the ipsilateral thyroid gland or the glandular girdle extends to the base of the neck, forming a sinus or an incomplete fistula. Most of the children only have one internal port and no external opening. The inner mouth showed no penetration with the neck skin, while the outer oening occurred due to repeated infection or incision and drainage. The domestic and foreign literature reports suggested that CPSF can cause repeated inflammatory masses in the neck, acute suppurative thyroiditis and/or abscess formation more commonly on the left side, and rarely on the right.^6^, ^7^, ^8^ The current cause of this is unknown, and it might be due to the asymmetric development of bilateral fourth zygomatic arch. Some scholars believed that it is caused by the effect of right iliac arch without the influence of the vagus.^9^ Of the 21 patients, 14 in this study showed abscesses around the thyroid gland, and 1 had around righ (1/14), which was consistent with the previous literature. In this group of patients, 7 were aged less than 4 years and showed abscess in the parapharyngeal space or posterior pharyngeal space. Due to lack of experience, CPSF was not considered, and thus the internal fistula was not explored and cauterized. Symptoms recurred after performing a simple incision and drainage of the abscess. As a result, patients were hospitalized again and diagnosed by surgical exploration. This suggested that in clinical work, it is necessary to consider the possibility of CPSF in children with a deep neck abscess to avoid misdiagnosis.

Regarding the diagnosis of CPSF, in addition to detailed medical history, clinical characteristics, and local signs, it is necessary to integrate with relevant auxiliary examinations, including gastrointestinal angiography, endoscopy, ultrasound, enhanced CT and MRI. Lipiodol imaging, which are practical and low cost, showed the presence and shape of fistulas. However, they are influenced by edema around the mouth during inflammation period, and noncooperation of infants when swallowing contrast agent. The contrast agent cannot pass through the fistula, and had a development rate of only 50%–80%.^10^ Electronic laryngoscopy is difficult to expose the deep, concealed pear-shaped fossa. Some scholars used the modified Killian method to improve the diagnostic rate. However, it is affected by age, shape and operators’ skill, which is less applicable for children. CTs and ultrasound examinations of the neck are frequently used in children. Typical signs in CT images showed abnormal density of the skin in piriform fossa-thyroid region or anterior cervical region, with a possibility of air-containing cavities, thyroid morphology changes or ring-gap enlargement.^11^ Ultrasound is a common examination method for diagnosing neck abscess in children due to its simple, non-invasive features and children’s easier cooperation. It also can be used as a significant auxiliary examination for diagnosing PSF.^11^ In the enrolled patients, 7 patients had air shadow or gas-liquid equilibrium in the deep neck and 11 patients demonstrated inflammatory swelling in the middle and lower anterior medial portion of the left sternocleidomastoid muscle, accompanied by gas shadows. Ten cases displayed a gas echo in the upper left thyroid region or a fistula that sneaked from the posterior anterior thyroid. Therefore, children with deep neck abscess who have gas accumulation or gas-liquid equilibrium revealed by CT or ultrasound are highly suspected as a proof of existence of CPSF.

All 21 patients with deep neck abscess had drainage under general anesthesia. At the same time, the piriform fossa was examined under self-retaining laryngoscope during operation, and low temperature plasma instrument was given during the inflammatory period to cauterize the internal fistula. There are some controversies about the treatment of endoscopic and peripheral mucosa by physical or chemical methods as adhesions and scars are formed, leading to closure of the internal orifice. Some scholars considered that this method is inaccurate and relapses easily, and recommended that it is the choice to completely remove the fistula after an acute infection period.

However, the complete resection of the fistula which also is traumatic to children, requires exquisite operational skills. Also, the operation is rather complicated with a series of postoperative sequelae, including vocal cord paralysis (3.7%～33.0%), neck scar deformity, etc.^12^ With less trauma and quick recovery in children, more and more scholars have chosen endoscopic surgical treatment, and achieved good results in recent years.^13^, ^14^, ^15^ Children with CPSF have a short disease course, small internal fistulas, and short sinuses (compared to adults), which are also an advantage of endoscopic cauterization. Nieoucar reviewed the recurrence rate of various treatments, in which the recurrence rate of simple incision drainage was 89%, open fistula resection was 15%, endoscopic cauterization was 15%, and partial thyroid resection in the open surgery of the neck was 8%. Liang^16^ reported laser ablation of 19 cases of CPSF, the first rate of fistula closure was 89.4% (17/19), and the second rate was 94.7% (18/19). The cumulative closure rate after 3 times of laser ablation was 100%. Dong^17^ analyzed 146 CPSF patients who underwent endoscopic low temperature ablation, with a recurrence rate of 15.1%. Twenty two patients developed hoarseness, and were recovered after 0.5–6 months of follow-up. In our study, after 1 year follow-up, no recurrence occurred. Compared with laser surgery, low temperature plasma has two advantages: firstly, the temperature of the plasma cutter head is relatively low (40°～70 °C), which reduces the damage to the mucosa and recurrent laryngeal nerve surrounding the piriform fossa to some extent; secondly, low temperature plasma is easy to operate, especially in deep, angled situations. Therefore, compared with surgical trauma, postoperative complications and recurrence rate, hypothermia treatment in the piriformis is a better choice.

Regarding the timing of surgery, some scholars believed that surgeries during an inflammatory period increases the risk of bleeding, and even affects the closure of fistula, but others suggest that endoscopic cauterization can be performed simultaneously by using sensitive antibiotics. In this study, endoscopic cauterization was performed in patients when they were in inflammatory period. The postoperative recovery remained good without any hoarseness or hemorrhage. This suggests that incision and endoscopic cauterization simultaneously can be used to reduce the risk of multiple anesthesia and negative effects on children.

However, there are several limitations in our study. Firstly, it involves a retrospective study design, small sample size, short observation time and findings from a single medical center. Secondly, selection of hypothermia treatment of piriform fossa, the duration of ablation of internal fistula, time interval, head temperature, extent and depth of the effect of postoperative efficacy requires further clinical research. Further studies including multicenter research, long observation and comparison of treatments and characteristics in different periods of children are warranted.

## Conclusion

According to this retrospective study, CPSF is an important cause of deep neck abscess in children. CPSF is often overlooked by clinicians, but should be used as one of the most important differential diagnosis of neck mass clinically. If pus cavity gas-liquid equilibrium or air shadow is suggested by neck-enhanced CT or cervical ultrasound examination, it is highly possible that a fistula existed. It is recommended to establish drainage and use self-retaining laryngoscopy to confirm the diagnosis. After that, low temperature plasma can be performed to cauterize and close the internal fistula with the assistance of an endoscope.

## Funding

This work was supported by the Healthy Department Projectof Zhejiang Province (2018KY452). The authors have no otherfunding, financial relationships, or conflicts of interest to disclose.

## Conflicts of interest

The authors declare no conflicts of interest.
